# Sex Differences in Antennal Transcriptome of *Hyphantria cunea* and Analysis of Odorant Receptor Expression Profiles

**DOI:** 10.3390/ijms25169070

**Published:** 2024-08-21

**Authors:** Weichao Ma, Yaning Li, Lina Yang, Shanchun Yan

**Affiliations:** 1 School of Forestry, Northeast Forestry University, Harbin 150040, China; maweichao@nefu.edu.cn (W.M.);; 2 Key Laboratory of Sustainable Forest Ecosystem Management-Ministry of Education, Northeast Forestry University, Harbin 150040, China

**Keywords:** *Hyphantria cunea*, antennal transcriptome, sex difference, odorant receptor expression level

## Abstract

Insects rely on olfaction for mating, finding oviposition sites, and locating hosts. *Hyphantria cunea* is a serious pest that severely damages forests. Differential expression analysis of olfactory-related genes between males and females is the basis for elucidating the functions of olfactory-related proteins in *H. cunea*. In this study, Illumina HiSeqTM 4000 high-throughput sequencing technology was used to perform transcriptome sequencing of the antennal tissues of adult male and female *H. cunea*. Functional annotation was conducted using the NR, Swiss-Prot, KOG, KEGG, and GO databases, and the results showed that the antennal transcriptome of adult *H. cunea* contained 50,158 unigenes. Differential expression analysis identified 3923 genes that were significantly differentially expressed between male and female antennae. A total of 221 olfactory-related genes were annotated, and 96 sex-biased genes were identified, including 13 odorant receptors (ORs), 48 odorant binding proteins (OBPs), 7 chemosensory proteins (CSPs), 10 ionotropic receptors (IRs), 10 sensory neuron membrane proteins (SNMPs), 2 gustatory receptors (GRs), and 6 odorant-degrading enzymes (ODEs), indicating that there were differences in olfaction between male and female *H. cunea*. Quantitative real-time PCR was used to verify the expression levels of 21 putative general odorant receptor genes in male and female antennae. *HcunOR4* and *HcunOR5* showed female-biased expression; *HcunOR48*, *HcunOR49* and *HcunOR50* showed male-biased expression. The results were consistent with the transcriptome differential analysis. The screening of male-biased odorant receptor genes might provide a theoretical basis for the functional characterization of odorant receptors for recognizing sex pheromones in *H. cunea*.

## 1. Introduction

Insects rely on their olfactory senses to perceive various odors and chemical cues in the environment, enabling them to locate mates, find hosts, identify oviposition sites, and evade predators [1,2]. The olfactory sensing process of insects recognizing odor molecules and elicit behavioral responses involves two primary steps: (1) In the peripheral olfactory system, odor molecules enter the lymph through the pores of the antennae and are transported by olfactory-related proteins to the dendritic membranes of olfactory neurons, subsequently activating membrane-bound olfactory-related proteins; (2) olfactory neurons convert the chemical signals into electrical signals, transmitting them to the central nervous system, eliciting behavioral responses in insects [3,4]. Chemosensory proteins (CSPs) and odorant binding proteins (OBPs) are involved in dissolving odor molecules and transporting them to the vicinity of olfactory neuron dendritic membranes. Odorant receptors (ORs) and ionotropic receptors (IRs) on the dendritic membranes recognize odor molecules and transmit signals to the central nervous system. After the olfactory response, odorant-degrading enzymes (ODEs) and sensory neuron membrane proteins (SNMPs) degrade odor molecules, preventing continuous stimulation of odor receptors that could damage the insect’s nervous system [5]. The insect antennal transcriptome serves as an important foundation for studying the function of olfactory proteins. In recent years, with the development of high-throughput sequencing technologies, an increasing number of insect antennal transcriptomes have been reported [6,7].

*Hyphantria cunea* is a highly polyphagous pest that can feed on a wide range of host plants, including almost all forest trees and even crops, except for some hardwood and evergreen coniferous trees [8]. *H. cunea* has a large feeding capacity, with larvae exhibiting the ability to withstand starvation and strong adaptability. In addition, *H. cunea* has a broad dispersal range and can be transmitted through multiple pathways, causing severe economic losses [9]. The antennal transcriptome of *H. cunea* was first reported in 2016, identifying olfactory-related proteins such as OBPs, CSPs, ORs, SNMPs, GRs, and IRs [10]. In addition, they performed semi-quantitative and relative quantitative analysis of the expression levels of 27 OBPs and 17 CSPs in different tissues between the two sexes, but sex-biased expression levels of odorant receptor genes were not identified. Since the publication of the antennal transcriptome of *H. cunea* in 2016, only a few studies have been published on the function characterization of general odorant receptors and sex pheromone-binding proteins in *H. cunea* [11,12,13]. However, the identification of odorant receptors for recognizing the sex pheromones of *H. cunea* has not been reported. This suggests that there may be difficulties in the identification of sex pheromone receptors in *H. cunea*. The sex pheromones of *H. cunea* belong to type II moth sex pheromones, which are relatively rare [14]. Thus, the odorant receptors for recognizing sex pheromones of *H. cunea* may have low identity with the odorant receptors that recognize the more common type I moth sex pheromones [15,16]. Therefore, it was hypothesized that the sex pheromone receptors of *H. cunea* may not belong to the traditional sex pheromone receptor evolutionary branch. Analyzing the sex-biased expression of the antennal transcriptome and the differential expression of odorant receptor genes of adult *H. cunea* may help to screen for male-biased odorant receptor genes, which could provide a theoretical basis for the identification of the sex pheromone receptors in *H. cunea*.

This study utilized second-generation transcriptome sequencing technology and fluorescent quantitative PCR technology to analyze the differences in transcription levels in the antennal tissues of *H. cunea*. A total of 221 olfactory-related genes were annotated, and in the differential analysis, 96 sex-specific genes were identified, including 13 ORs, 48 OBPs, 7 CSPs, 10 IRs, 10 SNMPs, 2 GRs, and 6 ODEs, indicating the presence of olfactory differences between male and female *H. cunea*. Both GO enrichment analysis and KEGG enrichment analysis were conducted, and the differentially expressed genes were mainly enriched in the subcategories of single-organism process (GO) and global and overview maps (KEGG). Fluorescent quantitative PCR technology was used to analyze the relative expression levels of 21 general odorant receptor genes obtained from the evolutionary analysis of the antennae of both male and female moths. *HcunOR4* and *HcunOR5* showed female-biased expression, while *HcunOR48*, *HcunOR49*, and *HcunOR50* exhibited male-biased expression. These results were consistent with the transcriptome differential analysis, and the identification of genes with male-biased expression provides a foundation for predicting the sex pheromone receptors of *H. cunea*.

## 2. Results

### 2.1. Quality Control

The filtering results of the raw transcriptome data of *H. cunea* antennae are shown in Table 1 and Table S1. The number of raw reads (raw data) from the F-An-1 biological replicate was 38,038,376, with the number and percentage of high-quality reads (clean reads) based on raw reads being 37,905,884 (99.65%) and the number of low-quality reads being 132,050 (0.35%). The percentage of high-quality data of the raw data of biological replicate M-An-2 was the lowest, at 99.62%. After filtering the data, the analysis of the base composition and quality distribution was performed, as shown in Table 1 and Table S1. Taking the biological replicate F-An-1 as an example, the total number of bases in the raw data was 5,705,756,400 (in bp), and the total number of high-quality bases after filtering was 5,685,746,323 (in bp). The number of bases with a sequencing quality reaching above the Q20 level (clean bases) after filtering and its percentage of raw bases was 5,566,314,157 (97.90%). The number of bases with a sequencing quality reaching above the Q30 level after filtering and its percentage of raw bases (or clean bases) was 5,315,695,472 (93.49%). The number and percentage of bases with N bases in the single-end read after filtering was 70,126 (0.00%). The GC ratio of the filtered sequence bases was 2,421,542,093 (42.59%). The lowest Q20 value was found in the biological replicate M-An-3 (97.75%), and the lowest Q30 value was in the biological replicate M-An-3 (93.20%), both of which were above 89%.

### 2.2. Sample Relationship Analysis

**Figure 1:**
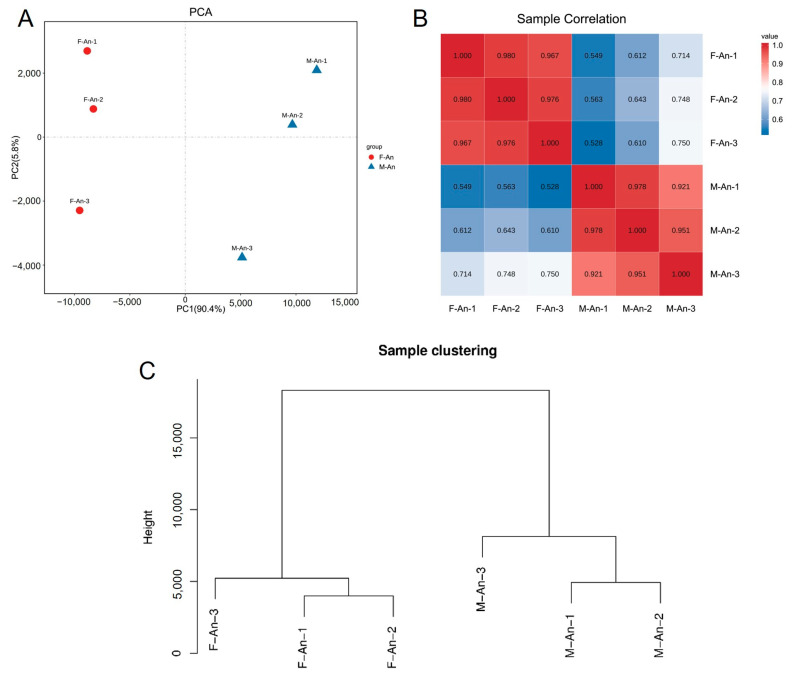
Sample relationship analysis. (**A**) Principal component analysis between female and male antennae samples; (**B**) correlation analysis of individual biological replicates in males and females; (**C**) hierarchical clustering between biological replicates of male and female antennae tissues.

The results of the principal component analysis (PCA) are shown in Figure 1A; male and female samples are clearly separated by PC1 (*x*-axis) which accounts for 90.4% of the variation, indicating small differences between the biological replicates of each group and good reproducibility. Considering the PCA values of each group’s biological replicates, it was evident that the reproducibility of the three female biological replicates was better than that of the male group (Figure 1A). Referring to the filtered read length (reads) and base-derived data, it was apparent that the M-An-3 deviated from the M-An-1 and M-An-2 groups. Taking the expression levels of any two groups, the Pearson correlation coefficient between each pair of samples was calculated. The lowest correlation coefficient in the female (F-An-X) group was between the F-An-1 and F-An-3 groups, with a coefficient of 0.967, while in the male (M-An-X) group, it was between the M-An-1 and M-An-3 groups, with a coefficient of 0.921. This indicated that the samples in the female group had a higher correlation, and overall, the samples within each group exhibited high correlation and good reproducibility (Figure 1B). In the sample clustering analysis, all gene expression levels of the samples were analyzed to cluster the relationships between the samples. The Euclidean distance was represented on the *y*-axis, where a lower position indicated smaller differences between samples. The results indicated that the female and male samples clustered on two separate branches, with M-An-1 and M-An-2 samples showing smaller differences between biological replicates and the F-An-1 and F-An-2 samples being more similar. Additionally, the reproducibility between the female samples was higher than that of the male samples (Figure 1C).

### 2.3. Analysis of Annotation Results

According to the results of functional annotation using the NR, Swiss-Prot, KOG, KEGG, and GO databases for the transcriptome of the adult antennae of *H. cunea*, as shown in Table 2, there were a total of 50,158 unigenes. Among these, 41,287 genes (82.31%) were successfully annotated in at least one database. The highest number of annotated genes was found in the NR database, with 41,271 genes (82.28%) for both female and male adult antennae transcriptomes. By contrast, the GO database had the lowest number of annotated genes, with 8287 genes (17.69%). Additionally, there were 8871 genes (17.69%) that were not annotated in any of the databases.

### 2.4. Overall Statistics of Differential Genes

**Figure 2:**
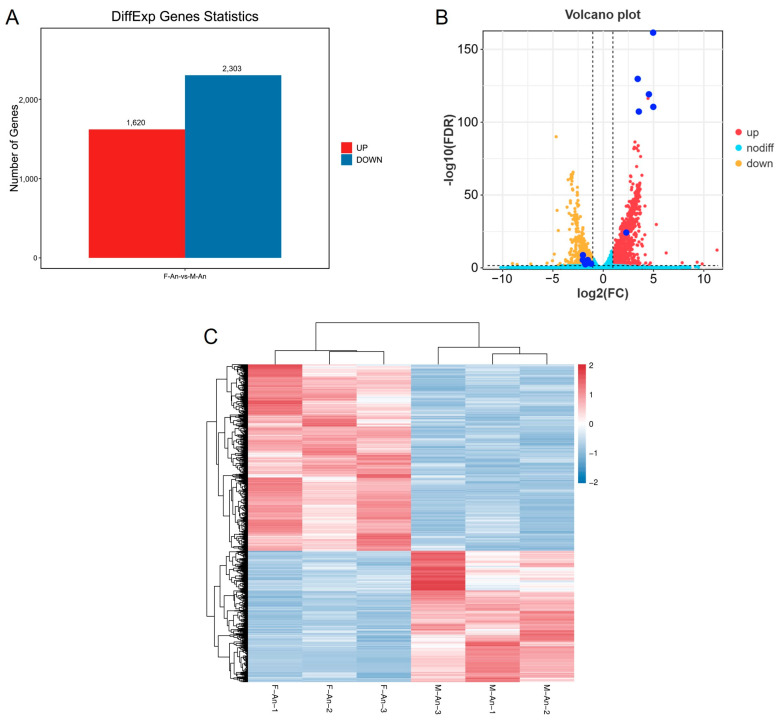
(**A**) Differential gene statistical map. (**B**) The volcano map of genes for sex difference of *H. cunea* (female vs. male); the dark blue dots are OR genes with significant DGE. The dashed lines mean FDR < 0.05 or |log2FC| > 1. (**C**) DGE heat map for gene expression clustering.

According to the differential analysis results, genes with FDR < 0.05 and |log2FC| > 1 were selected as significantly differentially expressed genes. A total of 3923 genes were identified as significantly differentially expressed in the antennae of female and male adults. Among these, 1620 genes were upregulated in female moths compared with male moths, while 2303 genes were downregulated (Figure 2).

### 2.5. GO Enrichment

**Figure 3:**
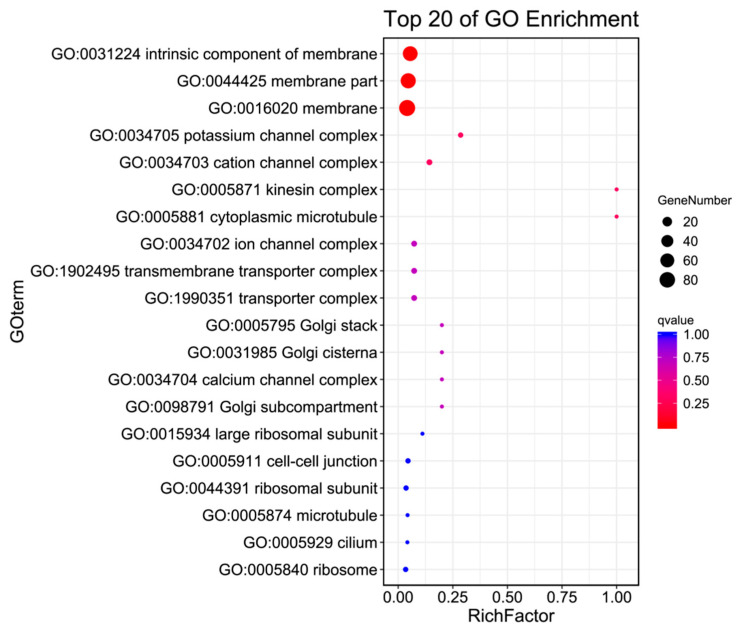
GO enrichment bubble plot of the antennal transcriptome of *H. cunea* (females vs. males).

The GO annotation of the antennal transcriptome of *H. cunea* included three main categories and 41 subcategories(as shown in Figure 3 and Table 3). In Biological Processes, there were 19 subcategories, including localization, reproductive processes, multicellular organismal processes, reproduction, and single-organism processes. Compared with males, females had 77 upregulated and 45 downregulated genes in the localization subcategory. In the single-organism process subcategory, females had 63 upregulated and 81 downregulated genes relative to males. In Molecular Function, there were 10 subcategories, including transporter activity, nucleic acid binding transcription factor activity, molecular transducer activity, electron carrier activity, and catalytic activity. Compared with males, females had 59 upregulated and 92 downregulated genes in the catalytic activity subcategory. In Cellular Component, there were 12 subcategories, including membrane part, membrane, extracellular matrix, supramolecular fiber, and cell junction. In these subcategories, compared with males, females had 50 upregulated and 38 downregulated genes in the membrane subcategory in the female category.

### 2.6. KO Enrichment

**Figure 4:**
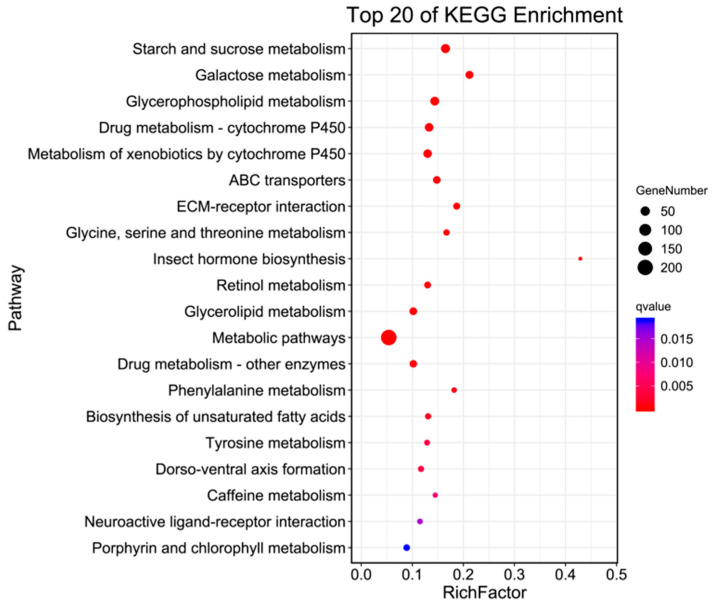
KO enrichment bubble plot of antennal transcriptome of *H. cunea* (female vs. male).

In living organisms, different genes coordinate with each other to carry out their biological functions. Pathway analysis, based on KEGG, a major public database for pathways, helped to further elucidate the biological functions of the genes. The enrichment circle presents the top 20 pathways (as shown in Figure 4), which were divided into three major categories: Metabolism, Environmental Information Processing, and Organismal Systems. Under Metabolism, there were 16 categories, including Starch and sucrose metabolism, Galactose metabolism, Glycerophospholipid metabolism, and Metabolism of xenobiotics by cytochrome P450, with 46, 31, 32, and 38 differentially expressed genes annotated in *H. cunea* antennal transcriptome, respectively. Environmental Information Processing included ECM-receptor interaction, ABC transporters, and Neuroactive ligand–receptor interaction, with 20, 27, and 11 differentially expressed genes annotated in the *H. cunea* antennal transcriptome, respectively. Organismal Systems, within the top 20 pathways, contained only one category, Dorso-ventral axis formation, with 14 differentially expressed genes annotated. Additionally, KO enrichment analysis annotated a total of 11,059 genes, out of which 1255 were differentially expressed (as shown in Table 4).

### 2.7. Identification of Candidate Olfactory-Related Genes

Through the Omicsmart platform of Genedenovo Biotechnology Co., Ltd. (Guangzhou, China), the results of the *H. cunea* antennal transcriptome were annotated. By searching and analyzing various keywords related to olfactory-related genes, a total of 221 candidate olfactory-related genes with complete open reading frames were identified from the *H. cunea* antennal transcriptome (as shown in Table 5).

The expression levels (FPKM values) of the annotated olfactory-related genes in males and females are respectively listed in Table 6 and Supplementary Tables S2–S7, along with whether there was differential expression between males and females and the length of the longest open reading frame. Among these, 26 ORs accounted for 11.76%, with a total of 13 significantly differentially expressed genes. Among these 13 significantly differentially expressed genes, 7 showed female-biased expression and 6 showed male-biased expression (Table 6. In addition, the DGE heat map for OR gene expression clustering is shown in Figure S1. There were 77 OBPs, accounting for 34.84%, with 48 significantly differentially expressed genes, 16 of which showed female-biased expression and 32 of which showed male-biased expression. There were 46 chemosensory protein genes CSPs, accounting for 20.81%, with a total of 7 significantly differentially expressed genes, 5 of which showed female-biased expression and 2 of which showed male-biased expression. Additionally, there were 3 GRs, accounting for 1.36%, with 2 significantly differentially expressed genes, both showing female-biased expression. Furthermore, there were 40 IRs, accounting for 18.10%, with a total of 10 significantly differentially expressed genes, 8 of which showed female-biased expression and 2 of which showed male-biased expression. There were 11 SNMPs, accounting for 4.98%, with 10 significantly differentially expressed genes, all showing male-biased expression. There were 18 ODEs, accounting for 8.14%, with a total of 6 significantly differentially expressed genes, all showing male-biased expression.

### 2.8. Protein–Protein Interaction Network Analysis

**Figure 5:**
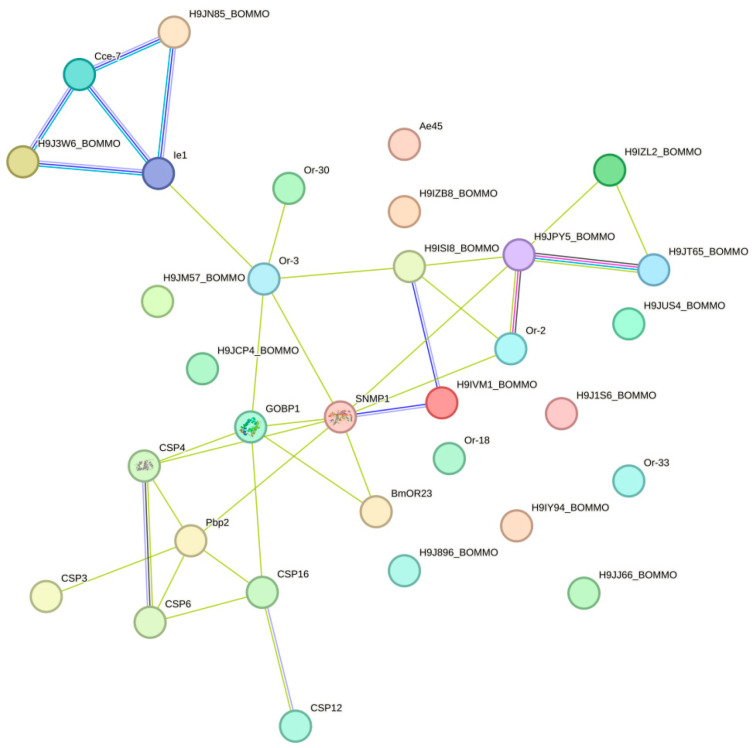
PPI network of DGE olfactory receptor genes.

A protein–protein interaction network analysis of 96 DGE olfactory receptor genes was performed using STRING (https://cn.string-db.org/ accessed on 6 August 2024). As *H. cunea* did not exist in the STRING database, there was only one option to use homologous proteins to map the interaction network. After comparing *Bombyx mori*, *Drosophila melanogaster*, and *Heliothis virescens*, *B. mori* was chosen as a reference and mapped protein interaction network. According to network statistical analysis shown in Figure 5, the network comprised 32 nodes, with an expected number of edges of 1, while the actual number of edges was 34. The protein–protein interaction (PPI) enrichment *p*-value was less than 1.0 × 10^−16^, indicating that the interactions among these proteins significantly exceeded random expectations. The average node degree of the network was 2.12, and the average local clustering coefficient was 0.386, suggesting the presence of a certain degree of locally dense connections within the network. SNMP1 served as a central node, interacting with multiple proteins, including GobP1, CSP4, Pbp2, CSP16, and CSP6, which might play important roles in signaling or sensory processes. CSP4, CSP8, CSP16, and CSP6 formed a tight cluster, demonstrating strong interactions between them, implying that they might collectively participate in sensory or defense mechanisms. Ie1 interacted with several proteins, including Cce-7 and H9J1W6_BOMMO, indicating a relatively active interaction profile within the network. Qrt8 interacted with Qrt2, Qrt30, GobP1, and other proteins of unknown function (such as H9J5Z_BOMMO), forming a larger subnet that suggests potential collaborative roles in a common biological process. In addition to its interaction with SNMP1, GOBP1 also interacted with proteins such as Qrt8 and Qrt2, which might be involved in sensory perception and signaling pathways.

### 2.9. Expression Levels of HcunOR Genes by RT-qPCR

**Figure 6:**
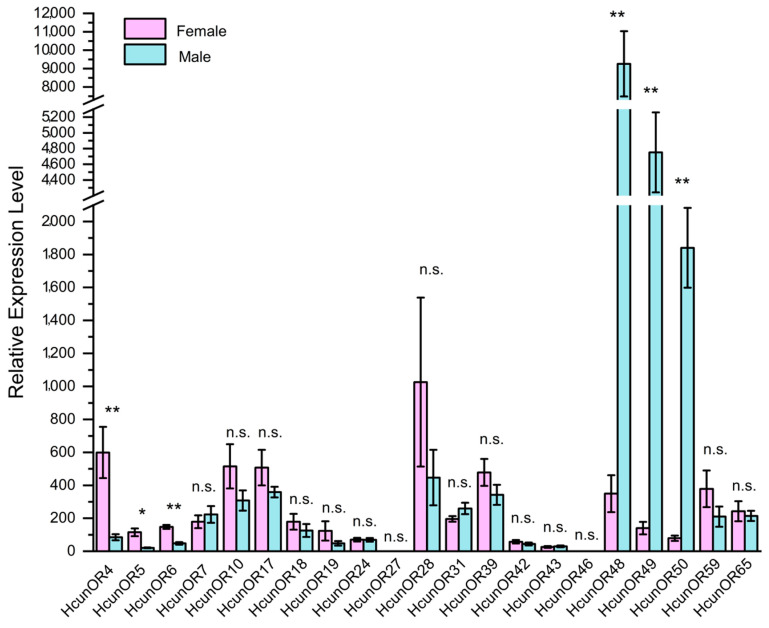
Expression profiles of general odorant receptors in the antennae of male and female *H. cunea* by qRT-PCR. **: *p* < 0.01, *: *p* < 0.05, n.s.: no significant difference.

On the basis of the transcriptome data annotation of 21 general odorant receptor genes in the antennae of *H. cunea* [13], quantitative real-time PCR (qRT-PCR) primers were designed to investigate the expression profiles of these 21 general odorant receptors in the antennae of both female and male moths. As shown in Figure 6, the expression patterns of these 21 odorant receptors in the female and male antennae were analyzed. Through independent sample *t*-tests, it was found that *HcunOR4* (*p* = 0.004 < 0.01), *HcunOR5* (*p* = 0.016 < 0.05), and *HcunOR6* (*p* = 0.002 < 0.01) were female-biased, while *HcunOR48* (*p* = 0.001 < 0.01), *HcunOR49* (*p* = 0.000 < 0.01), and *HcunOR50* (*p* = 0.000 < 0.01) were male-biased. The results were consistent with the differential expression analysis of the second-generation transcriptome data. For *HunOR4*, the average FPKM value in female antennae (F-An) was 1.23 ± 0.29, while in male antennae (M-An), it was 0.37 ± 0.11, with a *p*-value of 0.001. For *HcunOR5*, the average FPKM value in female antennae was 5.64 ± 1.23, and in male antennae, it was 1.42 ± 0.14, with a *p*-value of 3.44 × 10^−7^. For *HcunOR6*, the average FPKM value in female antennae was 1.58 ± 0.17, and in male antennae, it was 0.83 ± 0.18, showing higher expression in females, but the difference was not significant. For *HcunOR48*, the average FPKM value in female antennae (F-An) was 1.01 ± 0.04, while in male antennae (M-An), it was 32.72 ± 2.75, with a *p*-value of 6.56 × 10^−115^. For *HcunOR50*, the average FPKM value in female antennae (F-An) was 3 ± 0.29, and in male antennae (M-An), it was 71.85 ± 7.29, with a *p*-value of 9.18 × 10^−124^. For *HcunOR49*, the average FPKM value in female antennae (F-An) was 4.65 ± 0.51, and in male antennae (M-An), it was 149.07 ± 13.5, with a *p*-value of 1.47 × 10^−166^. In summary, the qRT-PCR results were consistent with the differential expression analysis of the transcriptome data. *HcunOR4* and *HcunOR5* were female-biased, while *HcunOR48*, *HcunOR49*, and *HcunOR50* were male-biased. *HcunOR6* did not show significant differential expression between the sexes in the transcriptome data.

## 3. Discussion

*H. cunea* is a polyphagous pest that seriously threatens the growth of forest trees. Conducting chemical ecology research on the *H. cunea* provides a theoretical basis for the scientific control of this pest. Although Zhang et al. published the transcriptome of *H. cunea* antennae in 2016, their analysis was based on second-generation transcriptome sequencing of mixed samples from male and female antennae [10]. As a result, the olfactory differences between male and female *H. cunea* remain unclear.

As high-throughput sequencing technology and bioinformatics technology have developed, transcriptome sequencing has become an important means of research in insect chemical ecology, and it is the basis for studying the molecular mechanism of insect olfaction [17,18]. In data quality control, the percentage of bases with a sequencing quality value of Q20 or higher (clean bases) in the raw data was at least 97.64%, and the percentage of bases with a sequencing quality value of Q30 or higher was at least 93.07%. Both were greater than 89%, indicating that the sequencing quality was good and the base composition was balanced with high quality. In the sample correlation analysis, the PCA analysis showed that the biological replicates of the female and male groups were separately clustered on both sides of the PC1 axis, and the cluster analysis of the sample relationships showed that the female and male samples were separately clustered on two branches; the lowest Pearson correlation coefficient between the two samples within each group was 0.921, indicating that the differences between the biological replicates within each group were small and the reproducibility was good [19].

Using GO enrichment analysis and KEGG enrichment analysis, the differentially expressed genes in the antennae of male and female *H. cunea* were enriched in specific GO categories or KEGG pathways to elucidate the differences in metabolism and gene functional classification in the adult *H. cunea* antennae. In the GO enrichment analysis, the differentially expressed genes in the antennae of male and female *H. cunea* were mainly enriched in the biological process category. Among them, 353 genes were female-biased and 406 genes were male-biased, indicating that the antenna function of males may be more complex. This was consistent with the appearance that the male *H. cunea* antennae were more complex and had more sensillum than the female antennae [20]. In the KEGG metabolic pathway enrichment analysis, a total of 977 differentially expressed genes were enriched in the metabolism category, followed by 127 differentially expressed genes enriched in the environmental information processing category. In the environmental information processing category, the main metabolic pathway subcategory was signal transduction (69 differentially expressed genes), which was consistent with the function of the antennae in converting chemical signals such as odors into electrical signals transmitted to the central nervous system [21,22,23]. This also indicated that there were differences in the function of the male and female antennae.

This study identified a total of 221 olfactory-related genes with complete open reading frames, including 26 odor receptor genes, 77 odorant binding protein genes, 46 chemosensory protein genes, 3 gustatory receptor genes, 40 ionotropic receptor genes, 11 neuronal membrane protein genes, and 18 odorant-degrading enzyme genes. In terms of total numbers, the quantity of olfactory-related genes identified in this study was similar to the results of antennal transcriptome annotation of other Lepidoptera insects, such as 238 candidate olfactory-related genes identified in the antennal transcriptome of the tea pest *Agriophara rhombata* [24], 142 genes identified in *Micromelalopha troglodyta* [25], and 217 genes identified in *Spodoptera litura* [26]. The number of odorant receptors was much lower than the 47 ORs identified by Zhang [10], and we inferred that this might be related to the depth of the third-generation transcriptome sequencing. This study was based on the third-generation sequencing data of our project team supplemented with second-generation transcriptome data, so it is possible that the depth was not sufficient and that some fragments were lost. In addition, this study, in combination with the genome of *H. cunea*, obtained 22 full-length odor receptor genes (about 400 amino acids), which was basically consistent with the approximately 400 amino acid odor receptor quantity (23 genes) reported by Zhang [10]. This study only identified three gustatory receptor genes, and it was speculated that this was related to the fact that the antennae are not the main taste organs. Several reports have indicated that gustatory receptors are mainly expressed in the mouthparts of insects [27]. There was a total of 96 differentially expressed olfactory-related genes, accounting for 43.24%, indicating differences in olfaction between male and female *H. cunea*. Olfactory binding protein and neuronal membrane protein genes with significantly differential expression were both male-biased in expression. Males rely on airborne sex pheromones to locate females when seeking mates, and accurate and sensitive localization requires the timely deactivation of each odor molecule [28]. Odor-degrading enzymes and sensory neuron membrane proteins both had the function of degrading odor molecules [29], and the overexpression of these two proteins in males could explain the above phenomenon.

In the published transcriptome paper on the antennae of *H. cunea*, Zhang used both semi-quantitative and relative quantitative methods to analyze the expression differences of 27 odorant-binding proteins and 17 chemosensory proteins between the two sexes. However, the expression differences of odorant receptors in the antennae of adult males and females were not analyzed. Therefore, this study used quantitative real-time PCR technology to verify the expression levels of the 21 annotated general odorant receptor genes in the two antennal tissues of males and females. Among them, six genes showed significant differential expression, with *HcunOR4*, *HcunOR5*, and *HcunOR6* preferentially expressed in females and *HcunOR48*, Hcun*OR49*, and *HcunOR50* preferentially expressed in males. Among the eight DGE OR genes in the transcriptome results, the quantitative results of *HcunOR4*, *HcunOR5*, *HcunOR48*, *HcunOR49*, and *HcunOR50* were consistent with the transcriptome differential analysis results. Three genes were inconsistent with the DGE results in the transcriptome. Although the transcriptome showed that *HcunOR6* was expressed at a higher level in females than in males, the difference was not significant, which might be related to the use of distinct biological replicates for transcriptome sequencing and quantitative analysis, sequencing errors, and variations in the methods used for differential significance analysis. Since the antennal transcriptome of *H. cunea* was reported, the functions of sex pheromone receptors in *H. cunea* have not been reported until now. The sex pheromones of *H. cunea* belong to the relatively rare type II sex pheromones in moths, with low structural similarity to the common type I sex pheromones. On the basis of the principle of receptor-ligand similarity [13,30], it was inferred that the odorant receptors for recognizing the sex pheromone of *H. cunea* might not belong to the traditional sex pheromone receptor evolutionary branch. The sex pheromones of *Ectropis grisescens* and *E. obliqua* were both type II pheromones of moths. The olfactory transcriptomes of these two moths were analyzed, and a possible type II pheromone receptor evolutionary branch was reported by analyzing the expression differences and evolutionary analysis of OR in the antennae of male and female adults, including four odorant receptors separately—EgriOR24, 31, 37, 44 and EoblOR24, 31, 37, 44—that were highly expressed in the antennae of males [31,32]. In this research, HcunO48, 49, and 50 were also highly expressed in male antennae and clustered together. Therefore, the screening of the overexpressed odorant receptors in male *H. cunea* could provide a basis for the screening of candidate sex pheromone receptors. The researchers successfully identified the sex pheromone receptors by screening the overexpressed odorant receptor genes in male *Spodoptera litura* and *Epiphyas postvittana*, and further verifying them through heterologous expression [33,34].

## 4. Materials and Methods

### 4.1. Insect Rearing and Tissue Collection

The *H. cunea* larvae were reared until pupation, and the pupae were sexed. Adult males and females aged 1–3 days post-eclosion were selected. Using fine forceps, antennae were excised from the base and placed in RNase-free centrifuge tubes. Three biological replicates were performed for both males and females, with 50 pairs of antennae in each replicate, and stored at −80 °C.

### 4.2. RNA Extraction and cDNA Library Construction

The preparation of transcriptome RNA samples involved extracting total RNA from the antennae of *H. cunea* using the Trizol (Invitrogen, Carlsbad, CA, USA) method. RNA quality was assessed on an Agilent 2100 Bioanalyzer (Agilent Technologies, Palo Alto, CA, USA) and checked using RNase-free agarose gel electrophoresis. Subsequently, poly(A)-containing eukaryotic mRNA was enriched using magnetic beads with oligo(dT) probes, while prokaryotic mRNA was enriched by removing rRNA with the Ribo-ZeroTM Magnetic Kit (Epicentre, Madison, WI, USA). Using the fragmented mRNA as a template and random oligonucleotides as primers, the first-strand cDNA was synthesized in the presence of M-MuLV reverse transcriptase. This was followed by the degradation of the RNA strand using RNase H and the synthesis of the second cDNA strand using DNA Polymerase I and dNTPs. Then, the cDNA fragments were purified with a QiaQuick PCR extraction kit (Qiagen, Venlo, The Netherlands), end-repaired, poly(A)-added, and ligated to Illumina sequencing adapters. The ligation products were size-selected by agarose gel electrophoresis, PCR-amplified, and sequenced using Illumina HiSeqTM 4000 by Gene Denovo Biotechnology Co. (Guangzhou, China).

### 4.3. Data Quality Control

To ensure data quality, a series of filtering steps was performed on the raw data prior to information analysis to reduce potential interference from invalid data. Initially, fastp was used to conduct quality control on the raw reads, resulting in the extraction of clean reads by filtering out low-quality data [35]. This included the removal of reads containing adapters, those with an N ratio exceeding 10%, reads composed entirely of A bases, and low-quality reads (where bases with a quality value Q ≤ 20 accounted for over 50% of the entire read). Following data filtration, an analysis of the composition and quality distribution of bases before and after filtering was conducted to visually demonstrate the data’s quality status.

To objectively analyze the transcriptome differences between the antennae tissues of male and female *H. cunea*, with each group comprising 3 biological replicates and each replicate consisting of antennae tissues from 50 individuals, a principal component analysis (PCA) was conducted using R (http://www.r-project.org/, accessed on 2 October 2020) on the basis of expression level information. The Pearson correlation coefficient was calculated to analyze the correlation and sample clustering between the samples, clarifying the repeatability between sample groups and assisting in the identification of outlier samples. The higher the similarity between samples within each group, the greater the repeatability and reliability of the data. This approach utilized the concept of dimensionality reduction to study the distance relationships between the samples. By calculating the Pearson correlation coefficient for the expression levels between any two samples, the correlation between any pair of samples was visually displayed in the form of a heatmap, providing an intuitive representation of the relationships between any two samples. This analysis helped in understanding the relationships and similarities between the samples, aiding in the identification of patterns and clusters within the dataset.

### 4.4. Annotation

The third-generation transcriptome from this project team was used as the reference genome, and paired-end clean reads were mapped to the reference genome using HISAT2 2.1.0 [36] and other parameters set as a default. The mapped reads of each sample were assembled by using StringTie v1.3.1 [37,38] in a reference-based approach. To annotate the isoforms, isoforms were BLAST-analyzed against the NCBI non-redundant protein (Nr) database (http://www.ncbi.nlm.nih.gov, accessed on 11 October 2020), the Swiss-Prot protein database (http://www.expasy.ch/sprot, accessed on 11 October 2020), the Kyoto Encyclopedia of Genes and Genomes (KEGG) database (http://www.genome.jp/kegg, accessed on 12 October 2020), and the COG/KOG database (http://www.ncbi.nlm.nih.gov/COG, accessed on 12 October 2020) with the BLASTx program (http://www.ncbi.nlm.nih.gov/BLAST/, accessed on 13 October 2020) at an E-value threshold of 1 × 10^−5^ to evaluate sequence similarity with genes of other species. Gene Ontology (GO) annotation was analyzed by Blast2GO software (https://www.blast2go.com/, accessed on 14 October 2020) [39] with Nr annotation results of isoforms. Isoforms that were ranked among those with the top 20 highest scores and were no shorter than 33 HSPs (high-scoring segment pairs) were selected to conduct the Blast2GO analysis. Then, functional classification of isoforms was performed using WEGO software (https://wego.genomics.cn/, accessed on 14 October 2020).

### 4.5. Expression Statistics and Differential Expression Analysis

For each transcription region, an FPKM (fragment per kilobase of transcript per million mapped reads) value was calculated to quantify its expression abundance and variations using RSEM software (http://deweylab.github.io/RSEM/, accessed on 20 October 2020) [40]. The differential expression analysis was performed using DESeq2 software (https://bioconductor.org/packages/release/bioc/html/DESeq2.html, accessed on 5 November 2020) to analyze the read count data obtained from the analysis of gene expression levels [41]. The analysis mainly consisted of the following three parts: (1) normalizing the read count data, (2) calculating the probability (*p*-value) of hypothesis testing on the basis of the model, and (3) performing multiple hypothesis testing correction to obtain the false discovery rate (FDR) value. On the basis of the differential analysis results, genes with FDR < 0.05 and |log2FC| > 1 were selected as significantly differentially expressed genes.

### 4.6. GO and KO Enrichment Analysis

GO Enrichment Analysis involved mapping differentially significant genes to various terms in the Gene Ontology (GO) (http://www.geneontology.org/, accessed on 25 November 2020) database and calculating the number of differentially expressed genes associated with each term. This helped in obtaining a statistical summary of the number of differentially expressed genes linked to a specific GO function. By employing hypergeometric testing, one can filter out GO terms that are significantly enriched in the set of differentially expressed genes compared with the entire genomic background.

In KO (KEGG Orthology) enrichment analysis, the approach is similar, but it focuses on KEGG pathways [42]. By using hypergeometric testing, one can identify significantly enriched pathways in the set of differentially expressed genes compared with the entire genomic background. This process helps in determining the principal biochemical metabolic pathways and signaling pathways in which the genes are differentially expressed. The GO and KO enrichment bubble plots were drawn by R (http://www.r-project.org/, accessed on 3 December 2020).

### 4.7. Protein–Protein Interaction Network

The protein–protein interaction network was identified using STRING (https://cn.string-db.org/, accessed on 6 August 2024), which determined genes as nodes and interactions as lines in a network. First, the amino acid sequences of 96 differentially expressed olfactory receptors were utilized to perform a homology search against the STRING database for homologous proteins from *B. mori*. Using the codes of the homologous proteins from *B. mori*, the protein–protein interaction network diagram was constructed, in which the minimum required interaction score was 0.150.

### 4.8. RT-qPCR Validation

Total RNA was extracted from the antennal tissues, and the first-strand cDNA was synthesized using the TOYOBO ReverTra Ace qPCR RT Master Mix with the gDNA Remover Kit to remove genomic DNA (gDNA) and perform reverse transcription. The fluorescence quantitative PCR (qRT-PCR) primers were designed using the Prime Premier 5 software. The qRT-PCR reaction system (20 μL) included 10 μL of qPCR Mix, 0.4 μL of reverse primer (10 μM), 0.4 μL of forward primer (10 μM), 1 μL of cDNA, and 8.2 μL of nuclease-free water. The qRT-PCR program was set as follows: 98 °C for 2 min, followed by 40 cycles of 98 °C for 10 s, 60 °C for 10 s, and 68 °C for 1.5 min. Actin (NCBI accession number: MH678709) was used as the reference gene [43], and the relative expression levels of the target receptor genes in the antennal tissues of the two sexes were calculated using the 2^−ΔΔCt^ method. The primer sequences of all genes are shown in Table S8. Each group had 3 biological replicates, and each biological replicate had 3 technical replicates. The relative quantification of gene expression was performed using the 2^−ΔΔCT^ method, and an independent sample T-test was conducted using SPSS 26 software to analyze the differences in the relative expression levels of each gene between males and females.

## 5. Conclusions

In this study, the second-generation transcriptome of female and male antennal tissues of *H. cunea* was determined by high-throughput sequencing technology; the olfactory-related genes were annotated, and the differences between females and males were analyzed. Data quality control and analysis of inter-sample relationships showed that the transcriptome quality was good. A total of 221 olfactory-related genes were annotated, and a total of 96 sex-differentiated olfactory-related genes were identified in differential analysis, indicating that there were differences in the olfactory senses between male and female *H. cunea*. The relative expression levels of 21 general odorant receptor genes in male and female antennae were verified by qPCR, which was consistent with the results of differential analysis of the transcriptome. The identification of the male-biased odorant receptor gene of *H. cunea* may provide a basis for the analysis of the molecular mechanism of the recognition sex pheromone of *H. cunea*.

## Figures and Tables

**Table 1:** The summary data of antennal transcriptome of *H. cunea* adults.

Sample	Clean Reads (%)	Clean Bases (bp)	Q20 (%)	Q30 (%)	N (%)	GC (%)
F-An-1	37,805,884 (99.65%)	5,685,746,323	97.90%	93.49%	0.00%	42.59%
F-An-2	36,204,050 (99.67%)	5,430,451,779	97.88%	93.41%	0.00%	42.01%
F-An-3	35,361,300 (99.64%)	5,303,989,239	97.80%	93.26%	0.00%	42.08%
M-An-1	35,108,044 (99.69%)	5,266,066,509	97.81%	93.30%	0.00%	42.54%
M-An-2	38,852,812 (99.62%)	5,827,771,407	98.00%	93.78%	0.00%	42.45%
M-An-3	33,361,348 (99.66%)	5,004,070,064	97.75%	93.20%	0.00%	42.02%

Note: Clean Reads (%): The total number of high-quality reads and the percentage of raw reads; clean bases: the total number of bases in the filtered high-quality data; Q20 (%): The percentage of bases whose sequencing base mass values reached the level above Q20 of clean bases; Q30 (%): the percentage of bases whose sequencing base mass values reached the level above Q30 of clean bases; N (%): the percentage of N-bases in a single-ended read of clean bases. GC (%): the proportion of base GCs of the filtered sequence.

**Table 2:** The results of unigene annotation of the antennal transcriptome of *H. cunea*.

Database	Unigene/s	Percentage (%)
Annotated in NR	41,271	82.28%
Annotated in Swiss-Prot	31,169	62.14%
Annotated in KOG	28,779	57.38%
Annotated in KEGG	21,591	43.05%
Annotated in GO	8287	16.52%
Not annotated in any database	8871	17.69%
Annotated in at least one database	41,287	82.31%

**Table 3:** GO enrichment analysis (F-An vs. M-An).

GO Term (Level 1)	GO ID (Level 2)	GO Term (Level 2)	Differential Genes(s)
Biological Process	GO:0051179	localization	122
	GO:0022414	reproductive process	2
	GO:0032501	multicellular organismal process	26
	GO:0000003	reproduction	2
	GO:0040007	growth	1
	GO:0044699	single-organism process	144
	GO:0040011	locomotion	2
	GO:0048519	negative regulation of biological process	7
	GO:0023052	signaling	23
	GO:0002376	immune system process	1
	GO:0032502	developmental process	14
	GO:0048518	positive regulation of biological process	4
	GO:0051704	multi-organism process	3
	GO:0065007	biological regulation	54
	GO:0071840	cellular component organization or biogenesis	19
	GO:0050789	regulation of biological process	46
	GO:0050896	response to stimulus	28
	GO:0009987	cellular process	137
	GO:0008152	metabolic process	124
Molecular Function	GO:0005215	transporter activity	42
	GO:0001071	nucleic acid binding transcription factor activity	12
	GO:0060089	molecular transducer activity	15
	GO:0009055	electron carrier activity	1
	GO:0016209	antioxidant activity	2
	GO:0004871	signal transducer activity	6
	GO:0098772	molecular function regulator	5
	GO:0003824	catalytic activity	151
	GO:0005198	structural molecule activity	2
	GO:0005488	binding	124
Cellular Component	GO:0044425	membrane part	75
	GO:0016020	membrane	88
	GO:0031012	extracellular matrix	1
	GO:0099512	supramolecular fiber	1
	GO:0030054	cell junction	2
	GO:0044421	extracellular region part	1
	GO:0005576	extracellular region	1
	GO:0032991	macromolecular complex	25
	GO:0044422	organelle part	12
	GO:0043226	organelle	35
	GO:0005623	cell	52
	GO:0044464	cell part	52

**Table 4:** KO enrichment analysis (F-An vs. M-An).

KEGG A Class	Metabolic Pathway	Differential Genes(s)
Metabolism	Amino acid metabolism	82
	Carbohydrate metabolism	157
	Biosynthesis of other secondary metabolites	9
	Energy metabolism	16
	Global and overview maps	390
	Glycan biosynthesis and metabolism	11
	Lipid metabolism	101
	Metabolism of cofactors and vitamins	50
	Metabolism of other amino acids	28
	Metabolism of terpenoids and polyketides	7
	Nucleotide metabolism	25
	Xenobiotics biodegradation and metabolism	101
Organismal Systems	Sensory system	2
	Immune system	1
	Environmental adaptation	8
	Development	14
	Circulatory system	1
	Aging	14
Genetic Information Processing	Translation	21
	Transcription	9
	Replication and repair	2
	Folding, sorting and degradation	23
Environmental Information Processing	Signaling molecules and interaction	31
	Signal transduction	69
	Membrane transport	27
Cellular Processes	Transport and catabolism	55
	Cellular community—eukaryotes	1
	Cell motility	1

**Table 5:** Olfactory-related genes in the transcriptomes of *H. cunea*.

Olfactory-Related Genes	Number of Genes	Proportion
Odorant receptor (OR) genes	26	11.76%
Odorant binding protein (OBP) genes	77	34.84%
Gustatory receptor (GR) genes	3	1.36%
Ionotropic receptor (IR) genes	40	18.10%
Chemosensory protein (CSP) genes	46	20.81%
Sensory neuron membrane protein (SNMP) genes	11	4.98%
Odorant degrading enzyme (ODE) genes	18	8.14%

**Table 6:** Identification and differential expression analysis of odorant receptors in *H. cunea*.

Gene-ID	Accession Number	FPKM Value (Mean ± SE)		Whether Differential	Significance in qPCR	ORF
		F-An	M-An			
HcunOR28	PQ100433	2.06 ± 0.42	1.41 ± 0.37	No	n.s.	414 aa
HcunOR7	PQ100434	0.42 ± 0.05	0.53 ± 0.10	No	n.s.	395 aa
HcunOR19	PQ100435	3.81 ± 0.35	1.48 ± 0.37	Yes	n.s.	252 aa
HcunOR59	PQ100436	13.56 ± 2.47	17.25 ± 3.58	No	n.s.	416 aa
HcunOR27	PQ100437	5.53 ± 0.44	1.96 ± 0.34	Yes	n.s.	419 aa
HcunOR42	PQ100438	0.68 ± 0.19	0.42 ± 0.17	No	n.s.	444 aa
HcunOR65	PQ100439	5.30 ± 0.42	3.00 ± 0.25	No	n.s.	226 aa
HcunOR61	PQ100440	5.02 ± 0.52	2.23 ± 0.29	Yes	-	396 aa
HcunOR49	PQ100441	4.65 ± 0.51	149.07 ± 13.50	Yes	**	404 aa
HcunOR48	PQ100442	1.01 ± 0.04	32.72 ± 2.75	Yes	**	405 aa
HcunOR50	PQ100443	3.00 ± 0.29	71.85 ± 7.29	Yes	**	396 aa
HcunOR6	PQ100444	1.58 ± 0.17	0.83 ± 0.18	No	**	382 aa
HcunOR1	PQ100445	2.39 ± 0.09	0.70 ± 0.19	Yes	-	157 aa
HcunOR10	PQ100446	7.61 ± 0.83	5.00 ± 0.55	No	n.s.	390 aa
HcunOR39	PQ100447	4.76 ± 0.30	6.86 ± 1.67	No	n.s.	386 aa
HcunOR46	PQ100448	19.45 ± 3.58	10.20 ± 0.93	No	n.s.	424 aa
HcunOR31	PQ100449	3.92 ± 1.29	5.90 ± 2.93	No	n.s.	395 aa
HcunOR24	PQ100450	18.04 ± 2.21	12.23 ± 1.88	No	n.s.	399 aa
HcunOR17	PQ100451	15.18 ± 1.91	3.86 ± 0.74	Yes	n.s.	405 aa
HcunOR5	PQ100452	5.64 ± 1.23	1.42 ± 0.14	Yes	*	377 aa
HcunOR4	PQ100453	1.23 ± 0.29	0.37 ± 0.10	Yes	**	288 aa
HcunOR3	PQ100454	7.71 ± 0.56	92.16 ± 7.79	Yes	-	430 aa
HcunOR2	PQ100455	15.33 ± 0.21	169.69 ± 11.46	Yes	-	422 aa
HcunORCO	PQ100456	6.68 ± 1.00	33.73 ± 5.99	Yes	-	473 aa
HcunOR18	PQ100457	18.27 ± 3.14	18.20 ± 0.67	No	n.s.	398 aa
HcunOR43	PQ100458	1.70 ± 0.31	1.72 ± 0.53	No	n.s.	397 aa

Note: The criteria of “whether differential” were that FDR < 0.05 and |log2FC| > 1, **: *p* < 0.01, *: *p* < 0.05, n.s.: no significant difference. “-”were those ORs without real-time PCR results.

## Data Availability

Data are contained within the article and Supplementary Materials.

## Supplementary Materials

The following supporting information can be downloaded at: https://www.mdpi.com/article/10.3390/ijms25169070/s1.
