# Supplementary material for: Sex Differences in Antennal Transcriptome of Hyphantria cunea and Analysis of Odorant Receptor Expression Profiles
Source: Int J Mol Sci. 2024 Aug 21;25(16):9070. doi: 10.3390/ijms25169070 (PMC11354529; doi:10.3390/ijms25169070)
Supplement: Supplementary file 1 [file ijms-25-09070-s001.zip › ijms-3111505-supplementary.pdf]

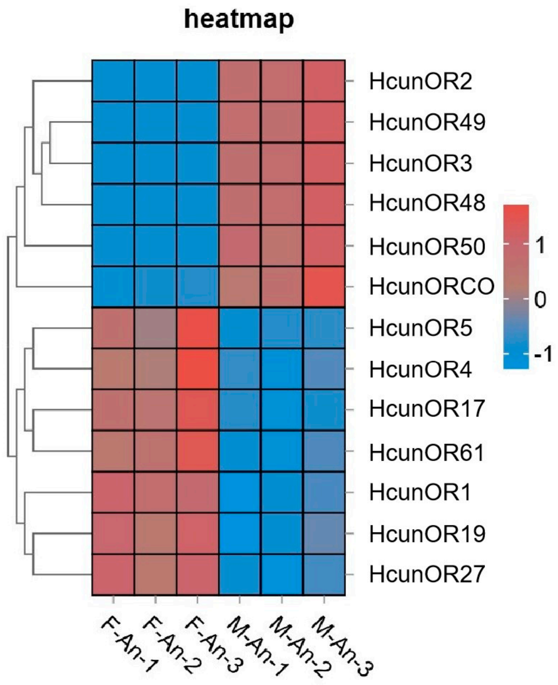

Figure S1 DGE heat map for OR expression clustering

Table S1 The statistics table of Raw Data of antennal transcriptome of *H. cunea* adults

Sample	Raw Reads	Raw Bases(bp)	Q20(%)	Q30(%)	N(%)	GC(%)
F-An-1	38038376	5705756400	97.78%	93.36%)	0.00%	42.59%
F-An-2	36323430	5448514500	97.77%	93.28%	0.00%	42.02%
F-An-3	35489090	5323363500	97.68%	93.12%	0.00%	42.08%
M-An-1	35218874	5282831100	97.71%	93.18%	0.00%	42.55%
M-An-2	39001170	5850175500	97.87%	93.63%	0.00%	42.46%
M-An-3	33476780	5021517000	97.64%	93.07%	0.00%	42.03%

Note: Raw Reads: The total number of raw reads; Raw Bases: The total number of bases before filtered; Q20(%): The percentage of bases whose sequencing base mass values reached the level above Q20 of Raw Bases; Q30(%): The percentage of bases whose sequencing base mass values reached the level above Q30 of Raw Bases; N(%): The percentage of N-bases in a single-ended read of Raw Bases. GC (%): The proportion of base GCs of the unfiltered sequence.

Table S2 Identification and differential expression analysis of odorant binding proteins in *H. cunea*

Gene-ID	Accession number	FPKM value (mean±SE)		Whether differential	ORF
		F-An	M-An		
HcunOBP1	PQ114163	248.51±29.57	168.55±5.17	No	137 aa
HcunOBP2	PQ114164	8.08±0.78	74.91±11.16	Yes	111 aa
HcunOBP3	PQ114165	14.04±2.08	150.74±10.59	Yes	166 aa
HcunOBP4	PQ114166	60.23±9.14	716.42±87.48	Yes	172 aa
HcunOBP5	PQ114167	8.03±1.38	85.94±3.34	Yes	207 aa
HcunOBP6	PQ114168	243.79±41.57	463.08±48.84	No	95 aa
HcunOBP7	PQ114169	145.5±8.69	77.34±4.69	No	146 aa
HcunOBP8	PQ114170	9.94±0.84	5.18±1.46	No	93 aa
HcunOBP9	PQ114171	335.4±11.37	183.10±17.22	No	103 aa
HcunOBP10	PQ114172	522.87±20	319.48±17.69	No	108 aa
HcunOBP11	PQ114173	7.18±0.45	4.76±1.22	No	100 aa
HcunOBP12	PQ114174	142.4±17.46	1315.69±181.37	Yes	123 aa
HcunOBP13	PQ114175	39.55±7.21	357.52±45.18	Yes	97 aa
HcunOBP14	PQ114176	68.92±10.16	662.64±92.16	Yes	144 aa
HcunOBP15	PQ114177	68.23±8.79	609.33±82.21	Yes	117 aa
HcunOBP16	PQ114178	115.92±10.28	923.24±125.93	Yes	123 aa
HcunOBP17	PQ114179	6.26±0.91	45.34±4.46	Yes	88 aa
HcunOBP18	PQ114180	68.47±6.09	401.00±55.02	Yes	72 aa
HcunOBP19	PQ114181	55.20±7.06	360.65±47.84	Yes	167 aa
HcunOBP20	PQ114182	105.81±6.96	63.52±0.98	No	137 aa
HcunOBP21	PQ114183	109.35±12.06	80.56±3.12	No	157 aa
HcunOBP22	PQ114184	189.66±18.85	130.52±8.47	No	161 aa
HcunOBP23	PQ114185	203.19±24.84	137.57±4.94	No	161 aa

HcunOBP24	PQ114186	2.61±0.40	0.96±0.17	No	77 aa
HcunOBP25	PQ114187	142.74±5.03	960.97±69.61	Yes	169 aa
HcunOBP26	PQ114188	188.22±7.73	1303.53±100.70	Yes	185 aa
HcunOBP27	PQ114189	51.83±6.59	342.28±41.36	Yes	159 aa
HcunOBP28	PQ114190	69.04±10.32	430.72±57.21	Yes	138 aa
HcunOBP29	PQ114191	198.04±18.32	132.70±4.29	No	164 aa
HcunOBP30	PQ114192	71.68±6.78	48.35±2.79	No	182 aa
HcunOBP31	PQ114193	183.53±18.55	123.49±6.44	No	161 aa
HcunOBP32	PQ114194	182.83±20.21	125.13±4.05	No	157 aa
HcunOBP33	PQ114195	202.53±21.26	139.48±4.06	No	163 aa
HcunOBP34	PQ114196	46.81±5.91	29.00±0.20	No	122 aa
HcunOBP35	PQ114197	34.86±0.91	12.65±1.78	Yes	102 aa
HcunOBP36	PQ114198	126.15±9.56	403.88±51.37	Yes	163 aa
HcunOBP37	PQ114199	203.95±20.48	61.00±5.02	Yes	115 aa
HcunOBP38	PQ114200	238.30±33.48	91.24±7.88	Yes	158 aa
HcunOBP39	PQ114201	13.73±0.95	150.51±16.74	Yes	104 aa
HcunOBP40	PQ114202	18.32±2.91	180.44±14.28	Yes	180 aa
HcunOBP41	PQ114203	69.87±10.69	714.06±99.61	Yes	136 aa
HcunOBP42	PQ114204	501.34±74.39	661.15±84.82	No	75 aa
HcunOBP43	PQ114205	233.79±48.91	52.36±6.34	Yes	121 aa
HcunOBP44	PQ114206	10.89±1.54	97.42±8.90	Yes	88 aa
HcunOBP45	PQ114207	194.51±25.77	836.93±67.34	Yes	141 aa
HcunOBP46	PQ114208	23.55±2.91	101.41±5.42	Yes	155 aa
HcunOBP47	PQ114209	332.29±53.71	322.00±10.43	No	145 aa
HcunOBP48	PQ114210	479.97±75.84	430.94±23.78	No	168 aa
HcunOBP49	PQ114211	459.09±49.18	352.38±9.73	No	162 aa
HcunOBP50	PQ114212	87.76±5.21	59.26±0.80	No	105 aa
HcunOBP51	PQ114213	453.19±38.36	66.73±6.21	Yes	116 aa
HcunOBP52	PQ114214	2609.15±224.74	557.25±6.52	Yes	143 aa
HcunOBP53	PQ114215	137.74±5.64	24.83±2.28	Yes	121 aa
HcunOBP54	PQ114216	200.11±13.37	769.56±39.96	Yes	110 aa
HcunOBP55	PQ114217	934.94±68.53	4729.11±519.89	Yes	139 aa
HcunOBP56	PQ114218	13.41±1.69	10.58±2.28	No	212 aa
HcunOBP57	PQ114219	1.76±0.120	1.20±0.17	No	183 aa
HcunOBP58	PQ114220	734.46±77.36	29.48±4.80	Yes	238 aa
HcunOBP59	PQ114221	116.79±5.65	29.61±1.80	Yes	234 aa
HcunOBP60	PQ114222	1543.82±176.19	395.17±66.65	Yes	133 aa
HcunOBP61	PQ114223	94.75±10.83	13.87±1.08	Yes	156 aa
HcunOBP62	PQ114224	132.14±1.97	48.34±9.77	Yes	145 aa
HcunOBP63	PQ114225	33.68±1.80	16.65±1.29	Yes	147 aa
HcunOBP64	PQ114226	96.04±14.32	378.37±34.32	Yes	141 aa

HcunOBP65	PQ114227	31.74±8.03	6.62±1.91	Yes	114 aa
HcunOBP66	PQ114228	51.53±8.82	433.34±53.06	Yes	86 aa
HcunOBP67	PQ114229	44.63±3.99	27.75±2.13	No	140 aa
HcunOBP68	PQ114230	5.39±0.11	13.22±1.18	Yes	79 aa
HcunOBP69	PQ114231	1.12±0.28	1.19±0.29	No	115 aa
HcunOBP70	PQ114232	67.56±12.01	143.41±19.74	Yes	140 aa
HcunOBP71	PQ114233	0.38±0.00	0.07±0.07	No	95 aa
HcunOBP72	PQ114234	19.65±1.98	11.61±5.82	No	137 aa
HcunOBP73	PQ114235	1306.72±58.30	464.26±38.69	Yes	222 aa
HcunOBP74	PQ114236	61.24±10.38	9.08±2.21	Yes	104 aa
HcunOBP75	PQ114237	181.63±32.74	57.79±5.11	Yes	166 aa
HcunOBP76	PQ114238	61.41±8.93	570.09±78.26	Yes	96 aa
HcunOBP77	PQ114239	0.00±0.00	0.00±0.00	No	79 aa

Table S3 Identification and differential expression analysis of ionotropic receptors in *H. cunea*

Gene-ID	Accession number	FPKM value (mean±SE)		Whether differential	ORF
		F-An	M-An		
HcunIR1	PQ100463	0.49 ± 0.11	0.38 ± 0.13	No	217 aa
HcunIR2	PQ100464	3.39±0.53	7.13±1.49	Yes	212 aa
HcunIR3	PQ100465	22.75 ± 2.24	30.69 ± 3.44	No	742 aa
HcunIR4	PQ100466	11.66 ± 1.56	17.97 ± 1.51	No	548 aa
HcunIR5	PQ100467	1.34±0.29	3.09±0.53	Yes	345 aa
HcunIR6	PQ100468	6.87 ± 0.56	4.95 ± 0.81	No	239 aa
HcunIR7	PQ100469	25.26 ± 3.86	13.96 ± 3.69	No	420 aa
HcunIR8	PQ100470	8.07±1.2	3.26±0.78	Yes	241 aa
HcunIR9	PQ100471	6.99 ± 1.48	3.61 ± 0.97	No	256 aa
HcunIR10	PQ100472	6.13±1.04	3.04±0.64	Yes	256 aa
HcunIR11	PQ100473	8.58 ± 1.38	4.59 ± 0.94	No	463 aa
HcunIR12	PQ100474	9.87±1.64	4.45±0.82	Yes	368 aa
HcunIR13	PQ100475	2.4 ± 0.43	2.16 ± 0.6	No	367 aa
HcunIR14	PQ100476	1.88 ± 0.15	1.05 ± 0.33	No	100 aa
HcunIR15	PQ100477	4.93 ± 0.5	3.1 ± 0.06	No	619 aa
HcunIR16	PQ100478	1.71 ± 0.03	0.92 ± 0.02	No	484 aa
HcunIR17	PQ100479	2.57 ± 0.48	2.2 ± 0.49	No	644 aa
HcunIR18	PQ100480	2.03 ± 0.25	1.59 ± 0.28	No	276 aa
HcunIR19	PQ100481	1.07 ± 0.28	1.41 ± 0.53	No	770 aa
HcunIR20	PQ100482	3.19 ± 0.44	2.38 ± 0.56	No	493 aa
HcunIR21	PQ100483	3.47 ± 0.62	2.68 ± 0.48	No	460 aa
HcunIR22	PQ100484	3.47 ± 0.55	2.79 ± 0.8	No	528 aa
HcunIR23	PQ100485	2.42 ± 0.37	1.85 ± 0.51	No	508 aa

HcunIR24	PQ100486	0.78 ± 0.2	1.07 ± 0.4	No	804 aa
HcunIR25	PQ100487	3.07 ± 0.69	2.28 ± 0.65	No	802 aa
HcunIR26	PQ100488	13.91±1.83	6.41±1.07	Yes	806 aa
HcunIR27	PQ100489	0.45 ± 0.13	0.44 ± 0.12	No	146 aa
HcunIR28	PQ100490	4.53±0.73	1.82±0.18	Yes	343 aa
HcunIR29	PQ100491	2.39 ± 0.69	1.29 ± 0.41	No	473 aa
HcunIR30	PQ100492	4.15±0.53	1.54±0.17	Yes	473 aa
HcunIR31	PQ100493	1.53±0.27	0.68±0.13	Yes	125 aa
HcunIR32	PQ100494	2.77 ± 0.2	2.24 ± 0.56	No	267 aa
HcunIR33	PQ100495	9.14±1.31	3.72±0.33	Yes	497 aa
HcunIR34	PQ100496	2.54 ± 1.06	1.49 ± 0.65	No	189 aa
HcunIR35	PQ100497	4.59 ± 1.17	2.25 ± 0.63	No	384 aa
HcunIR36	PQ100498	2.96 ± 0.62	1.58 ± 0.31	No	702 aa
HcunIR37	PQ100499	1.07 ± 0.15	1.37 ± 0.36	No	601 aa
HcunIR38	PQ100500	3.78 ± 0.39	3.27 ± 0.54	No	624 aa
HcunIR39	PQ100501	2.92 ± 0.29	2.75 ± 0.67	No	566 aa
HcunIR40	PQ100502	0.87 ± 0.18	1.34 ± 0.43	No	371 aa

Table S4 Identification and differential expression analysis of sensory neuron membrane proteins in *H. cunea*

Gene-ID	Accession number	FPKM value (mean±SE)		Whether differential	ORF
		F-An	M-An		
HcunSNMP1	PQ108675	2.05±0.39	11.81±2.07	Yes	192 aa
HcunSNMP2	PQ108676	71.18±8.13	230.73±10.34	Yes	315 aa
HcunSNMP3	PQ108677	78.23±1.47	262.94±38.64	Yes	519 aa
HcunSNMP4	PQ108678	22.52 ± 0.84	43.14 ± 7.72	No	238 aa
HcunSNMP5	PQ108679	37.69±1.90	107±8.84	Yes	310 aa
HcunSNMP6	PQ108680	4.45±0.98	30.02±4.9	Yes	394 aa
HcunSNMP7	PQ108681	3.21±0.63	18.65±2.08	Yes	316 aa
HcunSNMP8	PQ108682	3.93±0.81	31.6±7	Yes	474 aa
HcunSNMP9	PQ108683	5.10±0.90	33.6±6.18	Yes	525 aa
HcunSNMP10	PQ108684	2.81±0.38	16.02±2.16	Yes	179 aa
HcunSNMP11	PQ108685	21.63±0.18	57.48±8.68	Yes	245 aa

Table S5 Identification and differential expression analysis of chemical sensory proteins in *H. cunea*

Gene-ID	Accession number	FPKM value (mean±SE)		Whether differential	ORF
		F-An	M-An		
HcunCSP1	PQ111077	48.89 ± 6.04	47.94 ± 11.98	No	131 aa
HcunCSP2	PQ111078	71.88 ± 10.29	80.01 ± 18.05	No	127 aa

HcunCSP3	PQ111079	0.96 ± 0.07	0.75 ± 0.1	No	113 aa
HcunCSP4	PQ111080	5.37 ± 0.69	3.52 ± 0.58	No	316 aa
HcunCSP5	PQ111081	421.57±45.84	193.62±18.03	Yes	127 aa
HcunCSP6	PQ111082	264.22 ± 22.93	138.63 ± 8.97	No	146 aa
HcunCSP7	PQ111083	3264.2 ± 245.41	2060.23 ± 293.8	No	120 aa
HcunCSP8	PQ111084	144.36±8.97	52.41±4.68	Yes	88 aa
HcunCSP9	PQ111085	46.14 ± 1.31	32.86 ± 5.96	No	115 aa
HcunCSP10	PQ111086	19.9 ± 0.66	21.55 ± 2.53	No	128 aa
HcunCSP11	PQ111087	9.89 ± 0.29	10.9 ± 1.81	No	123 aa
HcunCSP12	PQ111088	475.7 ± 28.5	838.26 ± 161.19	No	125 aa
HcunCSP13	PQ111089	27.73 ± 2.4	21.13 ± 3.5	No	77 aa
HcunCSP14	PQ111090	74.51 ± 14.72	101.36 ± 13.29	No	126 aa
HcunCSP15	PQ111091	871.53 ± 51.27	968.49 ± 137.77	No	127 aa
HcunCSP16	PQ111092	28.13 ± 1.76	19.49 ± 2.08	No	105 aa
HcunCSP17	PQ111093	0.86 ± 0.11	0.63 ± 0.19	No	99 aa
HcunCSP18	PQ111094	6.44±0.77	17.36±1.61	Yes	289 aa
HcunCSP19	PQ111095	0.58±0.1	1.88±0.19	Yes	168 aa
HcunCSP20	PQ111096	58.43 ± 7.16	39.19 ± 2.14	No	124 aa
HcunCSP21	PQ111097	4.12±0.57	1.38±0.49	Yes	107 aa
HcunCSP22	PQ111098	0.3 ± 0.09	0.19 ± 0.05	No	87 aa
HcunCSP23	PQ111099	214.68 ± 14.01	164.92 ± 16.24	No	83 aa
HcunCSP24	PQ111100	0 ± 0	0 ± 0	No	93 aa
HcunCSP25	PQ111101	0.88 ± 0.28	0.77 ± 0.36	No	94 aa
HcunCSP26	PQ111102	226.93 ± 23.01	213.56 ± 30.69	No	97 aa
HcunCSP27	PQ111103	90.49 ± 8.11	78.89 ± 10.78	No	129 aa
HcunCSP28	PQ111104	81.77 ± 10.94	65.07 ± 7.3	No	128 aa
HcunCSP29	PQ111105	73.27±4.58	33.96±2.34	Yes	87 aa
HcunCSP30	PQ111106	73.96±5.86	36.72±3.77	Yes	81 aa
HcunCSP31	PQ111107	90.13 ± 7.5	76.11 ± 8.71	No	55 aa
HcunCSP32	PQ111108	139.64 ± 14.81	124.38 ± 13.73	No	128 aa
HcunCSP33	PQ111109	273.6 ± 26.31	284.4 ± 37.89	No	97 aa
HcunCSP34	PQ111110	394.68 ± 33.84	460.32 ± 66.08	No	128 aa
HcunCSP35	PQ111111	9.76 ± 1.89	10.74 ± 0.82	No	74 aa
HcunCSP36	PQ111112	336.81 ± 19.79	300.54 ± 35.19	No	81 aa
HcunCSP37	PQ111113	0.62 ± 0.16	0.73 ± 0.38	No	68 aa
HcunCSP38	PQ111114	0 ± 0	0 ± 0	No	68 aa
HcunCSP39	PQ111115	1.94 ± 0.37	1.79 ± 0.32	No	59 aa
HcunCSP40	PQ111116	23.02 ± 2.96	20.54 ± 1.83	No	55 aa
HcunCSP41	PQ111117	122.61 ± 17.87	126.53 ± 21.2	No	93 aa
HcunCSP42	PQ111118	2.38 ± 0.49	2.69 ± 0.66	No	64 aa
HcunCSP43	PQ111119	4.3 ± 0.42	4.11 ± 0.77	No	86 aa

HcunCSP44	PQ111120	10.29 ± 2.2	9.48 ± 1.47	No	125 aa
HcunCSP45	PQ111121	244.46 ± 14.24	239.27 ± 30.02	No	97 aa

Table S6 Identification and differential expression analysis of odorant-degrading enzymes in *H. cunea*

Gene-ID	Accession number	FPKM value (mean±SE)		Whether differential	ORF
		F-An	M-An		
HcunODE1	PQ108865	137.05±3.87	325.46±11.69	Yes	338 aa
HcunODE2	PQ108866	2.02 ± 0.46	1.5 ± 0.13	No	249 aa
HcunODE3	PQ108867	0.94±0.06	3.24±0.45	Yes	362 aa
HcunODE4	PQ108868	3.46 ± 0.23	2.22 ± 0.15	No	468 aa
HcunODE5	PQ108869	46.84 ± 10.01	33.21 ± 10.67	No	118 aa
HcunODE6	PQ108870	158.61 ± 35.6	108.51 ± 30.37	No	342 aa
HcunODE7	PQ108871	53.94 ± 12.9	37.94 ± 10.02	No	269 aa
HcunODE8	PQ108872	49.51 ± 17.94	25.67 ± 6.75	No	135 aa
HcunODE9	PQ108873	2.45±0.05	5.82±0.96	Yes	558 aa
HcunODE10	PQ108874	137.05±3.87	325.46±11.69	Yes	432 aa
HcunODE11	PQ108875	5.71±1.1	24.56±4.55	Yes	462 aa
HcunODE12	PQ108876	4.83±1.16	21.56±4.6	Yes	250 aa
HcunODE13	PQ108877	26.79 ± 3.1	28.24 ± 7.68	No	192 aa
HcunODE14	PQ108878	16.18 ± 2.26	14.29 ± 3.75	No	515 aa
HcunODE15	PQ108879	21.79 ± 3.38	18.31 ± 5.04	No	537 aa
HcunODE16	PQ108880	9.99 ± 1.6	9.9 ± 2.25	No	687 aa
HcunODE17	PQ108881	12.52 ± 0.79	11.47 ± 0.92	No	535 aa
HcunODE18	PQ108882	1.08 ± 0.32	0.77 ± 0.31	No	548 aa

Table S7 Identification and differential expression analysis of gustatory receptors in *H. cunea*

Gene-ID	Accession number	FPKM value (mean±SE)		Whether differential	ORF
		F-An	M-An		
HcunGR1	PQ108650	64.1±5.96	18.78±0.97	Yes	121 aa
HcunGR2	PQ108651	0.79±0.15	0.3±0.11	No	186 aa
HcunGR3	PQ108652	1.77±0.41	0.4±0.08	Yes	400 aa

Table S8 List of primers used in real-time PCR for *HcunORs*

Gene ID	Forward primer	Reverse primer
Actin	TCGACATCCGTAAGGACCTG	GTTGAGAGGGAAGCGAGGAT
HcunOR31	GTGACCACAAAGAAGTTATTCGTCA	GGCACAAAGCATCATGGAACAC
HcunOR18	CTATGGCTTTCGCTTTCCTCTA	GATAAAGAATAAAATGAGAATGACAGC
HcunOR5	CTGAGCGCTGGGGGAGTAG	TCGTTAAGGACGGCGAAGAT

HcunOR27	ACATATTGGAGGAAAAGGGAAGT	CCACGAAGCAAAGTGTTAAGGT
HcunOR19	ATCAAAAGATATCGACAAAGCGTT	ATTACACAAAAACAGCCCCAAGA
HcunOR6	GAGATGACACAACAGGGGGAAC	TCACCAGCAACGCTAAACTAAA
HcunOR46	ATTCAGAAACAAAGAGAAAAACACAT	GAAACCAGAAGAGGAAGAGAGTCAG
HcunOR59	CGTGGGCACAGAAGAATGAG	CTTGTAGTAAAAAGGTAGGCGGATA
HcunOR65	GCGAGGTTGTGGGGTCTAATA	CCTGCCAATAATCCTGGTGCTA
HcunOR39	TCAATGTGGTGCCGAGTTTC	CTGCTCCCTTAGCGTGTTTC
HcunOR17	CGCGTCTGCGAATCTGTTTAC	GTCAGTGGCACGATGAGGC
HcunOR48	TGGGTGGCAGAAATGTTCAG	TCAAATTAAGGATCCCGTAGGC
HcunOR4	GAGAGTTCCGGTGATGTTGTTACT	TGGCAAGTGCGCTAAAAGATC
HcunOR10	TATCGGAGTGGACAGTGTAGCC	CATTCGATCAGCAGTTTGTGATT
HcunOR50	TTAAGGCTTTCGTGGCTATGG	TTCGATCGGCTCAGGATATGT
HcunOR28	CCATGGTGTTCTGGTTGGTCA	TGTTTCATACATTGGTTCGAGACC
HcunOR7	CTGTACAAAACAAAAGAGAACGGG	GCTTGGATGATTTCAGAGGTTTC
HcunOR42	TACCGCCGCTGGTTTCAC	CCTCTTGTTGCTTTTGGCTTAG
HcunOR43	CAGTATTTGGGTGGGCTGTAAG	GTGCTGAACTCTGATTGATGTAGGT
HcunOR24	CAAATCGTCTTTTACGGGAA	CACCGCCACGAACCTATT
HcunOR49	AAGGCATCATTCGGATTGTTATT	TGTCGCTGTAGTAGCAAGGCAC
